# Automated Screening of Precancerous Cervical Cells Through Contrastive Self-Supervised Learning

**DOI:** 10.3390/life14121565

**Published:** 2024-11-28

**Authors:** Jaewoo Chun, Ando Yu, Seokhwan Ko, Gunoh Chong, Jiyoung Park, Hyungsoo Han, Nora Jeeyoung Park, Junghwan Cho

**Affiliations:** 1 Department of Biomedical Science, Graduate School, Kyungpook National University, Daegu 41944, Republic of Korea; jchana1996@knu.ac.kr (J.C.); yuando@knu.ac.kr (A.Y.); 2 Clinical Omics Institute, Kyungpook National University, Daegu 41405, Republic of Korea; stanleyhugo90@gmail.com (S.K.); gochong@knu.ac.kr (G.C.); hshan@knu.ac.kr (H.H.); 3 Department of Obstetrics and Gynecology, Kyungpook National University Chilgok Hospital, Daegu 41404, Republic of Korea; 4 Department of Pathology, Kyungpook National University Chilgok Hospital, Daegu 41404, Republic of Korea; jyparkmd@knu.ac.kr; 5 Department of Pathology, School of Medicine, Kyungpook National University, Daegu 41933, Republic of Korea; 6 Department of Physiology, School of Medicine, Kyungpook National University, Daegu 51944, Republic of Korea

**Keywords:** cervical cancer, cytology-based screening, distribution-augmented contrastive learning, self-supervised learning, precancerous cells

## Abstract

Cervical cancer is a significant health challenge, yet it can be effectively prevented through early detection. Cytology-based screening is critical for identifying cancerous and precancerous lesions; however, the process is labor-intensive and reliant on trained experts to scan through hundreds of thousands of mostly normal cells. To address these challenges, we propose a novel distribution-augmented approach using contrastive self-supervised learning for detecting abnormal squamous cervical cells from cytological images. Our method utilizes color augmentations to enhance the model’s ability to differentiate between normal and high-grade precancerous cells; specifically, high-grade squamous intraepithelial lesions (HSILs) and atypical squamous cells–cannot exclude HSIL (ASC-H). Our model was trained exclusively on normal cervical cell images and achieved high diagnostic accuracy, demonstrating robustness against color distribution shifts. We employed kernel density estimation (KDE) to assess cell type distributions, further facilitating the identification of abnormalities. Our results indicate that our approach improves screening accuracy and reduces the workload for cytopathologists, contributing to more efficient cervical cancer screening programs.

## 1. Introduction

Cytology-based screening is an effective method for detecting cancerous and precancerous lesions in the uterine cervix. This method has resulted in a significant worldwide reduction in both the incidence and mortality rates of cervical cancer. The 2020 International Agency for Research on Cancer (IARC) report stated that 80% of invasive cervical cancer cases are preventable, emphasizing the importance of early detection, HPV vaccination, and subsequent management through systematized cervical cancer screening programs [1].

Even though the advantage of cervical cytology is its ability to detect high-grade precancerous lesions [2], it still has limitations that must be addressed [3,4,5,6]. First, cytology screening has less sensitivity (50–70%). Second, it requires careful examination by well-trained cytopathologists, as the diagnosis depends on the observer’s experience. Third, intra- and inter-observer cytologic reproducibility is low. Additionally, each process is burdensome and labor-intensive. Finally, there is a shortage of trained experts despite increasing screening populations due to aging demographics. Recent developments in digital imaging and artificial intelligence (AI), particularly in deep learning (DL), are expected to revolutionize cancer screening, diagnosis, and treatment. This will improve the accuracy and reproducibility of image evaluations, and streamline the clinical workflows of surgical pathology and cytopathology [7,8,9,10,11]. In cervical cancer screening, a DL-based approach allows computer-aided diagnostic support systems to improve diagnostic accuracy and interpretability by allowing cytopathologists to concentrate on diagnosis and alleviate limited human resources. DL-based screening systems can also reduce financial costs and save time [8].

Many automated devices and recent computer-aided diagnostic models for cervical cytology have focused on detecting abnormal cells and proposing risk stratification or diagnostic classification, which require conventional cytologic examinations resulting in overflow workloads. Therefore, a new approach is needed to provide practical and efficient improvements and reduce the workloads of cytopathologic experts.

Previous approaches have used supervised learning methods that required extensive labeling to delineate cervical cells or regions of interest (ROIs) in a cytologic image [12,13,14]. More recently, unsupervised learning methods have been used to overcome the lack of labeled images, while self-supervised learning, a subclass of unsupervised models, can learn representative features from input images through proxy tasks such as contrastive learning [15] and clustering [16]. Self-supervised representation learning has been applied to one-class classification (OCC) tasks such as anomaly detection [17,18]. Although contrastive self-supervised models have shown improved performance in deep OCC [19], they may lack uniformity of representations [20]. Interestingly, distribution-augmented (DA) contrastive learning through data augmentation could expand the training distribution and overcome the uniformity limitation of one-class contrastive learning representations [17].

In this study, we applied a DA contrastive learning scheme to develop an OCC algorithm to learn the distribution of normal cervical epithelial cells interpreted as “Negative for Intraepithelial Lesion or Malignancy (NILM)” based on The Bethesda System (TBS) [21]. Using this technique, we can screen problematic cytology slides from NILM for further examination by a cytopathologic expert. We designed a two-step approach. First, representations of NILM-categorized cervical squamous cells are learned via DA schemes in an upstreaming task. A downstream analysis is then performed using the learned representations to investigate the efficiency of diagnostic accuracy and non-NILM/abnormal squamous cell localization. Since the color information of cervical squamous cells is useful in determining its clinicopathologic significance, we propose a color DA scheme in a simple framework for the contrastive learning of visual representations (SimCLR) [15] named DA-SimCLR. While color is useful, a model’s dependency on a single data source harms generalization when faced with a common problem in real-world clinics, batch effect [22] in images. Our proposed scheme takes the batch effect into account and shows diverse color distribution for generalization. The DA-SimCLR model was pretrained to learn representative features from NILM-categorized squamous cells. The feature representations were subsequently used for downstream analysis using kernel density estimation (KDE) [23], a one-class support vector machine (OC-SVM) [24], a Gaussian mixture model (GMM) [23], K-means clustering [25], and t-distributed stochastic neighbor embedding (t-SNE) [26].

## 2. Materials and Methods

### 2.1. Study Cohort

This study was approved by the Institutional Review Board of Kyungpook National University Chilgok Hospital (KNUCH IRB 2024-05-030-001). We searched the archives of medical records between January 2020 and November 2021 and collected 150 liquid-based cytology (LBC) slides from the cytology archives as an internal cohort.

This internal cohort included two groups based on the latest version of TBS for reporting cervical cytology. The first group was a normal class consisting of TBS diagnostic entities categorized as NILM (n = 100). The second group was an abnormal class comprising other entities interpreted as high-grade squamous intraepithelial lesions (HSILs) and “atypical squamous cells–cannot exclude HSIL” (ASC-H) (n = 50), all within the category of epithelial cell abnormalities [27]. However, this study did not include those designated as “atrophy”, which may mimic HSILs in low-power fields and often create sheets of parabasal cells that are confused with the syncytial variant of HSILs [21,28].

The Center for Recognition and Inspection of Cells (CRIC) cervix collection served as the external cohort dataset. This database comprised 400 images (with a resolution of 1376 × 1020 pixels) curated from conventional Pap smears, with a manual annotation of 11,534 cells. The annotations include the location of the nucleus center and classification labels based on TBS [21].

### 2.2. Overall Framework

**Figure 1:**
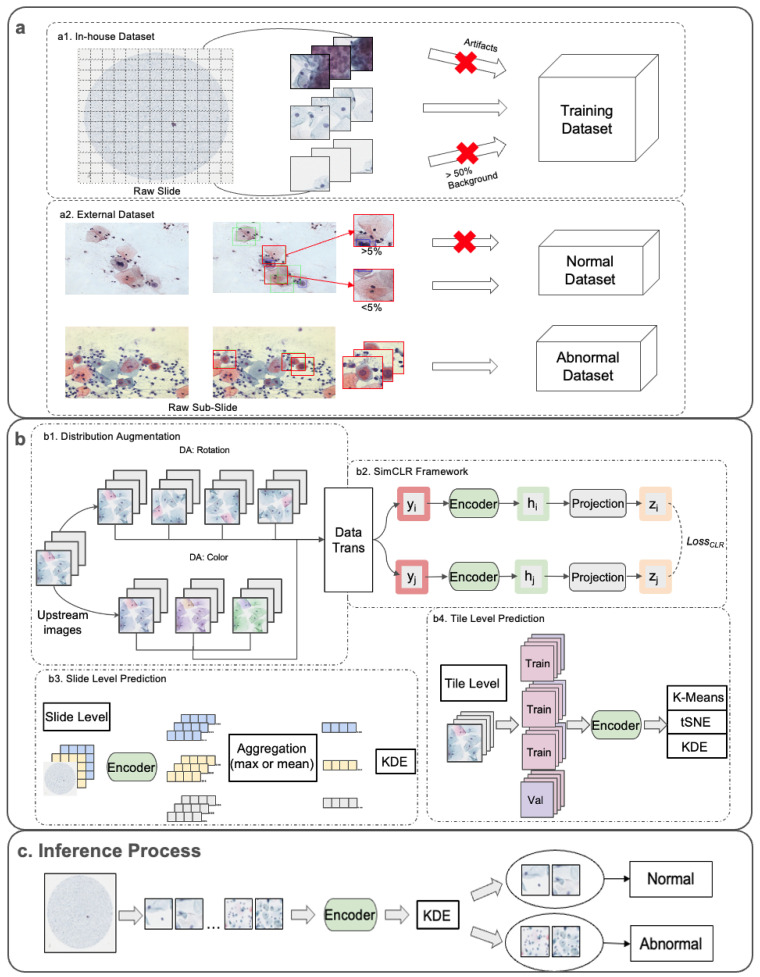
Overall framework of the experiment. (**a**) Illustration of dataset preparation for both (**a1**) in-house dataset where tiled images with artifacts and more than 50% background are excluded, and (**a2**) external dataset collection of normal and abnormal datasets, where the tiled images from the upper sub-slide are NILM-classified cells centered; the upper tiled image contains more than 5% of other classified cells so it is not added to the normal dataset. (**b**) Upstream tasks and downstream analysis in (**b1**) distribution augmentation of both color and rotation, (**b2**) SimCLR model framework with Resnet18 encoder, eight projection layers, and contrastive loss as upstream tasks, using the trained and frozen encoder; (**b3**) slide-level prediction is made using both types of aggregation methods, (**b4**) tile-level predictions by K-Means, t-SNE, and KDE. Images in downstream tasks (**b3**,**b4**) enter the trained encoder from (**b2**) without the distribution augmentation (**b1**) and the data trans (data transformation). (**c**) Slide images are tiled and immediately enter the encoder that was trained in (**b2**). The extracted image embeddings undergo trained KDE in (**b4**) for classification.

We designed a novel framework to distinguish abnormal (non-NILM, HSIL/ASC-H) from normal (NILM) squamous cervical cells. This pipeline consisted of dataset preprocessing, upstream pretraining, and downstream analysis tasks, as shown in Figure 1. Datasets were used for both the in-house and CRIC datasets [29] for external validation.

#### 2.2.1. Dataset Preprocessing

We preprocessed the raw data from our in-house and external CRIC datasets for input into the classification model. Each dataset was divided into abnormal (non-NILM, HSIL/ASC-H) and normal (NILM) squamous cervical cell classes.

For the in-house dataset, each LBC slide was scanned at 40× magnification (0.25 μm/pixel) using a digital slide scanner (Aperio GT450, Leica Biosystems Imaging, Inc., Vista, CA 92081, USA). All scanned images were downsampled by a factor of two (0.5 μm/pixel) and cropped into 256 × 256-pixel tiles, with each tile containing multiple cells. Tile images containing artifacts or more than 50% background were excluded (Figure 1(a1)).

In the external CRIC dataset, the manual annotations for the cell-level images were provided as rectangular boxes of 90 × 90 pixels, each containing a single cell. We cropped the raw sub-slide images (1376 × 1020 pixels) into 256 × 256-pixel tiles to ensure the cells were analyzed at the same tile size as the in-house dataset. For the abnormal class, we extracted tile-level images containing all HSIL and ASC-H cells (Figure 1(a2)). For the normal class, we selected tile images that contained less than 5% of regions annotated as abnormal, based on the rectangular box annotations (Figure 1(a2)). A total of 8374 tile images were created: 5746 normal and 2628 abnormal images. To balance the dataset, we randomly sampled 2628 normal images to match the 2628 abnormal images.

#### 2.2.2. Upstream Pretraining Tasks

In the upstream tasks, we built three pretrained models to learn representations of normal cervical cells: the generic (unmodified) SimCLR, DA-SimCLR (Color), and DA-SimCLR (Rotation). The DA-SimCLR (Color) and DA-SimCLR (Rotation) models were implemented by applying their respective distribution augmentation, as shown in Figure 1(b1).

The generic SimCLR without distribution augmentation schemes immediately entered the SimCLR framework with the upstream data. As shown in Figure 1b, the generic SimCLR framework [15] consisted of (1) data transformations, (2) an encoder as a feature extractor, (3) a multilayer perceptron (MLP)-based projection head, and (4) a contrastive loss function. The transformations included random resized crop, random horizontal flip, color jittering, random grayscale conversion, and Gaussian blur. A ResNet18 architecture was used for the encoder to extract representative features (h and h′) from the transformed images (y and y′). These extracted representations were then mapped to a lower-dimensional latent vector space via an MLP-based projection head. While the original SimCLR paper used one hidden layer, we employed eight hidden layers, which have shown to produce optimal results for deep OCC [17]. Also, the trained generic SimCLR encoder was used to train an MLP classifier with labels that served as a baseline.

#### 2.2.3. Downstream Analysis Approaches

Once the feature representations of normal cervical cells were learned from upstream works, we conducted subsequent downstream analysis using KDE [23], OC-SVM [24], GMM [23], K-means clustering [25], and t-SNE methods [26].

For anomaly detection methods, we used a non-parametric KDE to estimate distributions of a normal class from the representations learned by SimCLR. Since the KDE was trained by the encoded features from only the normal class of the training dataset, the KDE score indicated the likelihood value, representing the degree of belonging to the normal distribution. Furthermore, since the KDE was differentiable, we generated a Gradcam attention map [30] based on the KDE to provide a visual interpretation of where the model sees the given tile image. We also employed two other commonly used anomaly detection methods, OC-SVM and GMM, to validate the classifier’s performance. Unlike the non-parametric KDE, a parametric Gaussian mixture was used [17].

Since a normal label was given, we also performed a K-means clustering scheme to classify whether the sample data were normal or abnormal under semi-supervised settings. This algorithm works iteratively to assign each image data to one of K groups based on feature similarity. We observed that approximately four clusters were formed in the t-SNE graph using in-house data, so we used four clusters to calculate the K-means. We then defined the majority of each cluster as either normal or abnormal.

### 2.3. Experiments

**Figure 2:**
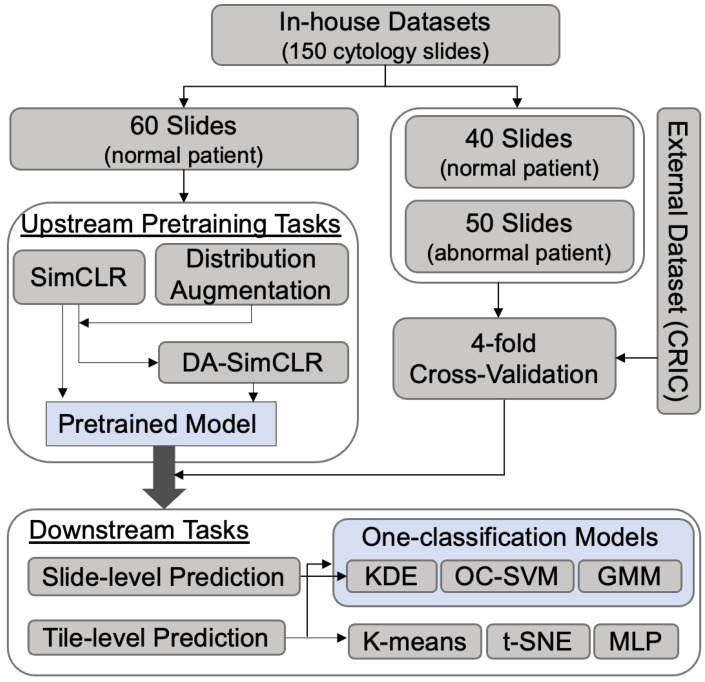
Flowchart of study design including dataset preparation and upstream/downstream analysis steps.

Our experiments consisted of dataset preparation, upstream pretraining tasks, and downstream tasks, as depicted in Figure 2. ChatGPT was used in the abstract and the conclusion of manuscript by inputting our manuscript (without the two) and further refining the ChatGPT’s drafts.

#### 2.3.1. Dataset Preparation

An in-house dataset was constructed consisting of 100 normal and 50 abnormal patients. From this dataset, 60 NILM cytology slides were used to train the generic SimCLR and DA-SimCLR models, representing the normal class. These models were trained in upstream tasks to learn the underlying features of normal cells using only NILM slides. We then performed downstream analysis for both slide-level and tile-level predictions. As shown in Table 1, 90 slides (40 normal and 50 abnormal, none of which were used in the upstream training) were utilized for slide-level prediction. For tile-level prediction, tile images were extracted from 52 slides (26 NILM normal class slides and 26 abnormal class slides). Each abnormal class image contained at least one or more abnormal cells classified as HSIL or ASC-H. In total, we extracted 1356 images of both normal and abnormal classes from their respective 26 slides. Each abnormal cell, including HSIL and ASC-H, was labeled with a bounding box by a professional pathologist. A total of 1586 abnormal cells were annotated from 1356 tile images and evaluated using the Intersection over Union (IOU) score. Table 1 summarizes the experimental dataset.

To further validate the robustness of our approach, we performed external validation using the CRIC dataset. This dataset was preprocessed to match the image sizes with our in-house data, as described in Section 2.2.1 (Dataset Preprocessing). All abnormal tile images were taken from CRIC and an equal number of randomly selected normal tile images were included to balance the dataset. Only tile-level predictions were made using the CRIC dataset, as whole-slide images were not provided. The in-house and CRIC tile-level prediction datasets were each divided into four folders, each containing 75% of the data for training and 25% for validation, enabling 4-fold cross-validation. The 75% training data were used to train K-means clustering, a MLP neural network for supervised learning, and OCC models for anomaly detection such as KDE, OC-SVM, and GMM. Only normal images from the training data were used in these models, except for the supervised learning. The remaining 25% of the data was used to assess performance.

#### 2.3.2. Upstream Pretraining Tasks

**Figure 3:**
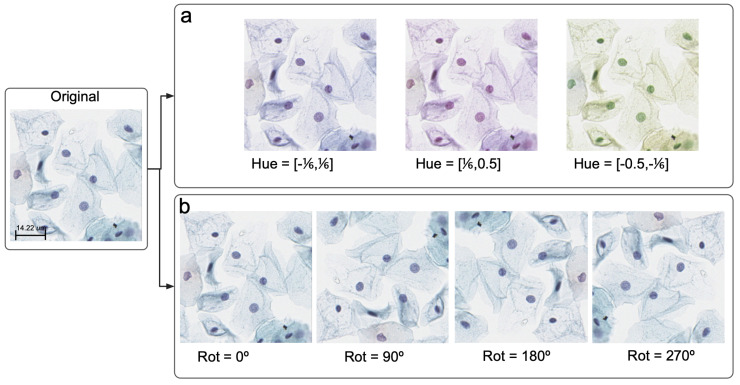
Representative examples of (**a**) color with three hue variations and (**b**) rotation transformation with four different angles (0, 90, 180, and 270 degrees) for distribution augmentation.

We trained three different models: basic SimCLR without augmentation; distribution augmentation (DA) with rotation, referred to here as DA-SimCLR (Rotation); and DA with color, referred to here as DA-SimCLR (Color). While the basic SimCLR model used no modifications or distribution augmentation, the DA models increased the dataset size threefold for DA-SimCLR (Color) and fourfold for DA-SimCLR (Rotation). DA-SimCLR (Color) created three additional datasets by applying color jitter transformations with the hue parameter ranges of ($\frac{-1}{6}$, $\frac{1}{6}$), ($\frac{-1}{2}$, $\frac{-1}{6}$), and ($\frac{1}{6}$, $\frac{1}{2}$), as shown in Figure 3a. DA-SimCLR (Rotation) generated four additional datasets by rotating the images by 0, 90, 180, and 270 degrees, as shown in Figure 3b. The color jitter strength was set to 0.4, while the other transformations used the default settings in PyTorch and PyTorch Lightning. However, DA-SimCLR (Color)’s transformation excluded the hue parameter from the color jitter. Examples of these distribution augmentations are shown in Figure 3.

Our experiment was conducted using the following settings: SGD [31] optimizer with a single cycle of cosine annealing learning rate scheduler [29], a learning rate of 0.01, a batch size of 32, temperature set to 0.2 (for contrastive loss), and a maximum of 100 epochs. All experiments were run on Nvidia RTX 3090 GPUs using the PyTorch and PyTorch Lightning frameworks. The upstream tasks with this setting took about 20, 58, and 82 h for SimCLR, DA-SimCLR (Color), and DA-SimCLR (Rotation) respectively.

#### 2.3.3. Downstream Tasks

Once trained, each model’s last epoch was chosen for the downstream analysis. The analysis, consisting of K-means clustering, t-SNE visualization, OC-SVM, GMM, and a KDE classifier, was conducted by a Scikit-learn library and evaluated by performance metrics (precision, recall, and F1 score) using a 4-fold cross-validation method. The area under the curve (AUC) score as a measure of classification performance was utilized and evaluation was conducted with the slide-level downstream dataset.

Slide-level diagnostics were performed as two different aggregation methods, mean and maximum. We took all tile representations from a single slide, and obtained the mean or maximum of each tile representation to produce the slide representation. As a result, 90 512-dimensional slide representations (40 normal and 50 abnormal slides) were produced and KDE was performed.

## 3. Results

### 3.1. In-House Tile-Level Prediction

**Figure 4:**
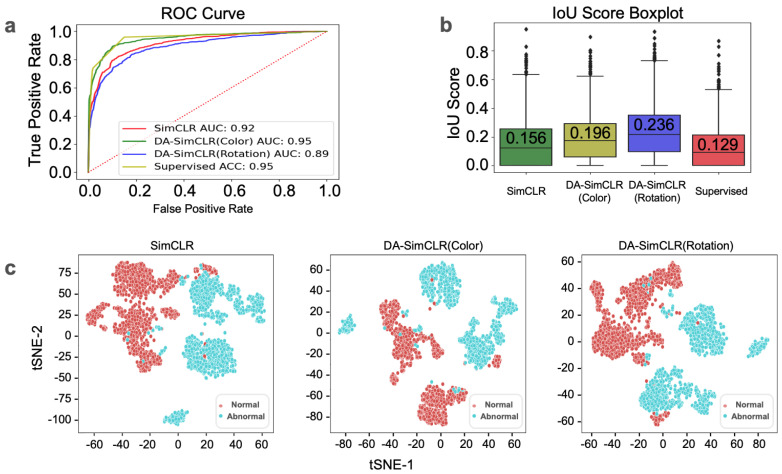
Downstream analysis results. (**a**) IOU score box plot of in-house tile level diagnostics with mean values. (**b**) ROC curve with KDE AUC of each model. (**c**) Embedding representation visualization using t-SNE.

After the upstream pretraining of three SimCLR models with 60 slides from the in-house dataset (Table 1), tile-level diagnostics were performed using the following methods for downstream analysis: K-means, t-SNE, KDE, and localization using Gradcam [30]. We observed classification results by the receiver operating characteristic (ROC) curve in Figure 4a with the four models. Of the models tested, the supervised method and the DA-SimCLR (Color) method performed best, followed by SimCLR and DA-SimCLR (Rotation). Figure 4b shows the overall IOU score, showing that DA models outperformed other models in localization. The t-SNE embedding is presented in Figure 4c, showing that all models seem to be separable. We observed a consistent trend of the F1 score, KDE, and OC-SVM yielding the best results for DA-SimCLR (Color), excluding the supervised method used solely as a reference, as shown in Table 2.

**Figure 5:**
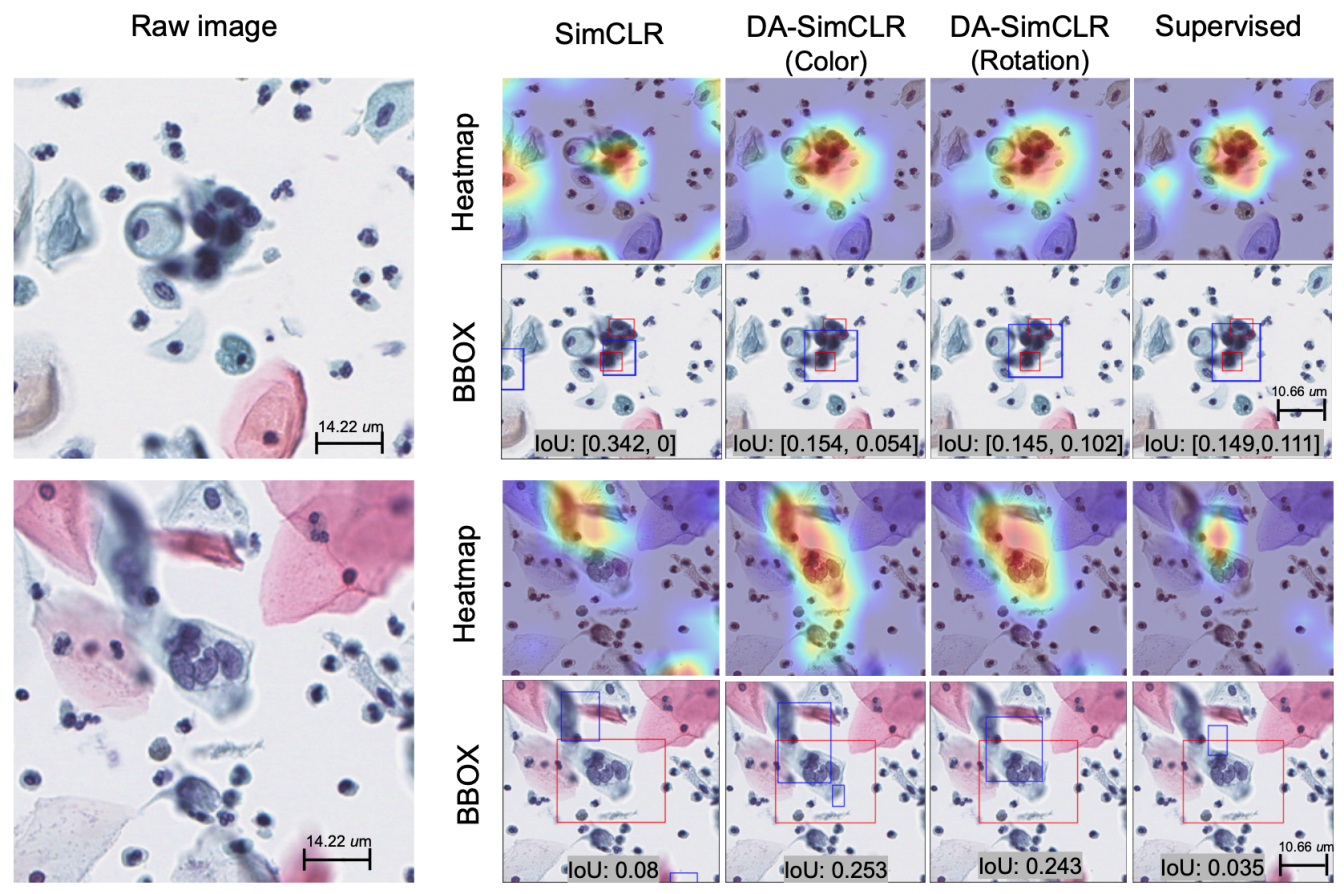
Two examples with heatmaps extracted from GradCAM, ground truth (red), and predicted bounding box (BBOX) images (blue).

Figure 5 presents two examples of abnormal cell localization by the predicted bounding box with heat map extracted from the GradCAM activation map. This visual explanation of OCC models highlights the abnormal cell regions, which were compared with a pathologist’s findings.

### 3.2. In-House Slide-Level Prediction

A slide-level diagnostic was performed using two different aggregation methods, mean and maximum. The KDE results based on the mean embeddings were AUC values of 0.898 for SimCLR, 0.879 for DA-SimCLR (Color), and 0.866 for DA-SimCLR (Rotation). The maximum values in the embeddings were an AUC of 0.947 for SimCLR, 0.956 for DA-SimCLR (Color), and 0.969 for DA-SimCLR (Rotation).

### 3.3. External Validation with CRIC Dataset

**Figure 6:**
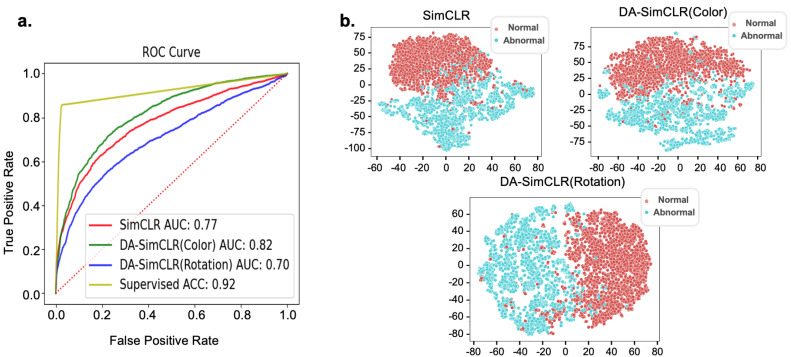
External dataset results. (**a**) ROC curve with respect to CRIC. (**b**) Embedding projected upon lower dimension through t-SNE.

For external validation, we utilized the same SimCLR model pretrained with our in-house dataset. Employing the three pretrained models, we conducted a downstream analysis using the K-means clustering classifier, OCCs (KDE, OC-SVM, and GMM), MLP supervised classifier, and t-SNE visualization. The supervised classifier outperformed other models, as shown in the ROC curve in Figure 6a, since all labels were given. With the exception of the supervised model, DA-SimCLR (Color) achieved the best AUC score with 0.817, followed by SimCLR and DA-SimCLR (Rotation). The tile embeddings obtained from the Resnet18 encoder of the pretrained model were projected on a t-SNE plot, as shown in Figure 6b. The t-SNE plot indicated that most of the abnormal tile embeddings from all models appeared to be highly separable, consistent with the K-means clustering classification results presented in Table 3. Among the models, DA-SimCLR (Rotation) achieved the highest F1 score, followed by SimCLR and DA-SimCLR (Color).

Similar to the in-house tile predictions, we observed a consistent trend where the OCC used on the pretrained DA-SimCLR (Color) model outperformed the other two (SimCLR and SimCLR (Rotation)) overall (see Table 3), leading to a notable difference in performance margins.

## 4. Discussion

We propose a distribution-augmented contrastive learning approach to screen abnormal squamous cervical cells from cytological images. Contrastive learning is a form of self-supervised learning algorithm that takes unlabeled images as representations extracted from the encoder. Similar image have embedding values that are closer together, while dissimilar image embeddings are farther apart. This type of learning is widely used for multi-class classification or transfer learning. Adaptations can be made to detect outliers according to the learned distribution under multi-class settings [32]. However, in real-world applications, the amount of data from certain classes can be much smaller than that of other classes.

For cervical cells, normal samples are more easily available than abnormal samples. Therefore, we enhanced our work from deep OCC [17], which is trained on only one normal class, to better detect outliers from the trained distribution. By exposing the model to vast variations of color, the model can become less sensitive to color. As proposed in [17], applying distribution augmentation to OCC makes inliers compact, therefore making outlier detection easier as outliers become more separable from the inliers. In that work, rotation was used as the distribution augmentation, but in our experiment, we applied color (more specifically, hue) as our distribution augmentation approach because color can be a critical factor in cytopathology images that affects the model’s decision-making.

**Figure 7:**
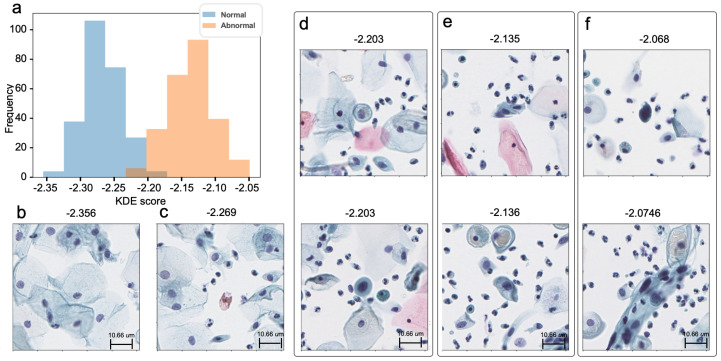
Image analysis on KDE scores. (**a**) KDE distributions; (**b**) a typical image sampled from KDE scores between −2.36 and −2.31; (**c**) an image sampled from scores of −2.27; (**d**) two representative images with KDE scores of −2.20; (**e**) two images within the score range of −2.14 to −2.13; and (**f**) two images within the range of −2.07 to −2.05.

The assessment of cervical cytology abnormalities used KDE to estimate a normal distribution. The range of abnormalities delineated by KDE scores is shown in Figure 7. Figure 7a–c illustrate typical examples of normal cytology, with predominantly normal cells. In the overlapping region of normal and abnormal scores within the KDE distribution, occasional abnormal cells were observed in certain tiles, as shown in Figure 7d. In contrast, atypical cytology, characterized by abnormally elevated KDE scores, consisted largely of abnormal cells, as shown in Figure 7e,f. Therefore, we can screen for abnormal squamous cervical cells using KDE distribution learned from only normal cytological images.

**Figure 8:**
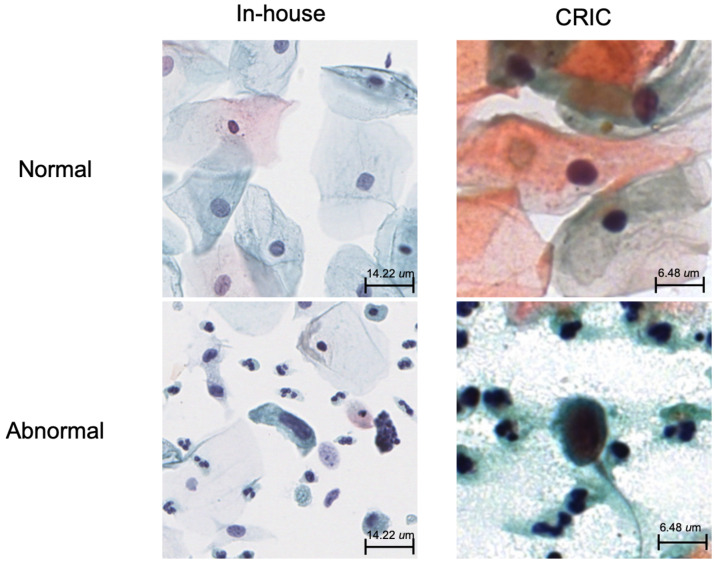
Dataset comparison of normal and abnormal tile samples of in-house (0.5 μm/pixel) and external datasets (0.226 μm/pixel).

Our proposed DA-SimCLR (Color) model outperformed the other tested models. When exposed to significant color distribution variation, DA-SimCLR (Color) became less sensitive to color information, resulting in improved generalization. The superiority of this method is evident in the results shown in Table 3 using the CRIC external dataset, which significantly differed from the in-house dataset, as shown in Figure 8. DA-SimCLR (Color) also achieved the best performance in most downstream metrics. The in-house dataset yielded comparable results, since the upstream pretrained model shared the same domain distribution. However, the external dataset exhibited a significant performance difference across models. This discrepancy between the in-house and external datasets stems from the strong color variations present in the external dataset.

There are limitations to our method. Although our approach is more adaptable to shifts in color distribution compared to other SimCLR models, a more advanced representation learning model, such as a cytology foundation model, offers a promising solution to address distribution shift issues in the dataset. Adapting a large-scale pretrained learning model and fine-tuning it with the target dataset is an alternative approach. Another limitation of our approach is the interpretation of decision-making based on cellular characteristics. For doctors and cytologists, cellular characteristics such as cell morphology, size, and nucleus number are important. However, our method primarily delivers results without emphasizing these features.

Extending this method to include cellular characteristics will be the focus of our future work. Previously, our research explored extracting features such as cell size, nucleus size, and morphology from tiled images [33]. Integrating these findings into our framework is a promising direction for future development. Furthermore, in this paper, we conducted validation with in-house and external datasets; however, compared to the amount of trained data, validation data were relatively low. To that end, multi-institute cohorts have provided us with thousands of slides that our model can be validated upon to confirm its effectiveness and reliability.

## 5. Conclusions

In this study, we introduced a distribution-augmented contrastive learning approach to effectively screen for abnormal squamous cervical cells from cytological images. Our method leverages self-supervised learning techniques, particularly focusing on color augmentations to enhance the model’s ability to discriminate between normal and abnormal cell types. The DA-SimCLR (Color) model demonstrated better performance across various metrics than other models, showcasing its adaptability to color distribution shifts, a common challenge in cytopathology.

The application of kernel density estimation (KDE) provided valuable insights into the distribution of cell types, facilitating the identification of abnormalities in cytological samples. The findings suggest that our framework has the potential to enhance cervical cancer screening programs, ultimately contributing to early detection and better patient outcomes.

## Figures and Tables

**Table 1:** Summary of experimental training and validation datasets.

		In-House Dataset		
		100 normal slides	50 abnormal slides	150 total slides
		(184,842 tiles)	(249,600 tiles)	(434,442 tiles)
Upstream Pretraining Tasks		60 slides	N/A	60 normal slides
		(96,646 normal tiles)		(96,646 normal tiles)
Downstream	Slide Level	40 normal slides	50 abnormal slides	90 slides
	Tile Level	1356 tiles	1356 tiles	2712 tiles
		(from 26 normal slides)	(from 26 abnormal slides)	
		External dataset (CRIC)		
Downstream	Tile Level	2628 tiles	2628 tiles	5256 tiles

**Table 2:** Downstream analysis results from the in-house dataset using the K-means clustering classifier, one-class classifier models (KDE, OC-SVM, and GMM), and the MLP supervised classifier on three pretrained SimCLR models.

	Downstream Analysis								
Upstream Pretrain Model	K-Means Clustering Classifier			One-Class Classification Models			Supervised Classifier		
	Precision	Recall	F1 Score	KDE	OC-SVM	GMM	GMM	Recall	F1 Score
SimCLR	0.92	0.95	0.93	0.92	0.94	0.97	0.96	0.95	0.95
	± (0.079)	± (0.072)	± (0.041)	± (0.10)	± (0.07)	± (0.02)	± (0.059)	± (0.053)	± (0.032)
DA-SimCLR	0.94	0.95	0.95	0.95	0.85	0.94			
(Color)	± (0.068)	± (0.019)	± (0.034)	± (0.05)	± (0.05)	± (0.05)			
DA-SimCLR	0.94	0.95	0.94	0.89	0.92	0.92			
(Rotation)	± (0.074)	± (0.045)	± (0.032)	± (0.14)	± (0.09)	± (0.10)			

**Table 3:** Downstream analysis results from the CRIC external dataset using the K-means clustering classifier, one-class classifier models (KDE, OC-SVM, and GMM), and the MLP supervised classifier.

	Downstream Analysis								
Upstream Pretrain Model	K-Means Clustering Classifier			One-Class Classification Models			Supervised Classifier		
	Precision	Recall	F1 Score	KDE	OC-SVM	GMM	Precision	Recall	F1 Score
SimCLR	0.83	0.91	0.87	0.60	0.77	0.40	0.98	0.86	0.91
	± (0.01)	± (0.05)	± (0.03)	± (0.07)	± (0.04)	± (0.09)	± (0.0082)	± (0.077)	± (0.047)
DA-SimCLR	0.85	0.74	0.78	0.74	0.82	0.61			
(Color)	± (0.02)	± (0.10)	± (0.06)	± (0.03)	± (0.01)	± (0.03)			
DA-SimCLR	0.83	0.94	0.88	0.51	0.70	0.40			
(Rotation)	± (0.01)	± (0.03)	± (0.01)	± (0.04)	± (0.03)	± (0.07)			

## Data Availability

The datasets presented in this article are not readily available due to privacy or ethical restrictions. Samples of preprocessed tiled data can be provided upon request to the corresponding authors.

## Abbreviations

The following abbreviations are used in this manuscript:

International Agency for Research on Cancer	IARC
Artificial intelligence	AI
Deep learning	DL
Regions of interest	ROI
One-class classification	OCC
Distribution-augmented	DA
Negative for Intraepithelial Lesion	NILM
The Bethesda System	TBS
Simple framework for contrastive learning visual representations	SimCLR
Kernel density estimation	KDE
One-class support vector machine	OC-SVM
Gaussian mixture model	GMM
t-distributed stochastic neighbor embedding	t-SNE
Liquid-based cytology	LBC
High-grade squamous intraepithelial lesion	HSIL
Atypical squamous cells–cannot exclude HSIL	ASC-H
Center for Recognition and Inspection of Cells	CRIC
Multilayer perceptron	MLP
Intersection over union	IOU
Area under the curve	AUC
Receiver operating characteristic	ROC
